# A conserved RNA structure is essential for a satellite RNA-mediated inhibition of helper virus accumulation

**DOI:** 10.1093/nar/gkz564

**Published:** 2019-07-03

**Authors:** Lu He, Qian Wang, Zhouhang Gu, Qiansheng Liao, Peter Palukaitis, Zhiyou Du

**Affiliations:** 1 College of Life Sciences and Medicine, Zhejiang Sci-Tech University, Hangzhou, Zhejiang 310018, China; 2 Department of Horticultural Sciences, Seoul Women's University, Nowon-gu, Seoul 01797, Republic of Korea

## Abstract

As a class of parasitic, non-coding RNAs, satellite RNAs (satRNAs) have to compete with their helper virus for limited amounts of viral and/or host resources for efficient replication, by which they usually reduce viral accumulation and symptom expression. Here, we report a cucumber mosaic virus (CMV)-associated satRNA (sat-T1) that ameliorated CMV-induced symptoms, accompanied with a significant reduction in the accumulation of viral genomic RNAs 1 and 2, which encode components of the viral replicase. In*trans* replication assays suggest that the reduced accumulation is the outcome of replication competition. The structural basis of sat-T1 responsible for the inhibition of viral RNA accumulation was determined to be a three-way branched secondary structure that contains two biologically important hairpins. One is indispensable for the helper virus inhibition, and the other engages in formation of a tertiary pseudoknot structure that is essential for sat-T1 survival. The secondary structure containing the pseudoknot is the first RNA element with a biological phenotype experimentally identified in CMV satRNAs, and it is structurally conserved in most CMV satRNAs. Thus, this may be a generic method for CMV satRNAs to inhibit the accumulation of the helper virus via the newly-identified RNA structure.

## INTRODUCTION

In recent years, long non-coding RNAs (lncRNAs) have received a great deal of attention as a new class of RNAs that function in numerous biological processes across all biological agents (1–6). Generally, endogenous lncRNAs are expressed in limited quantities in eukaryotic cells, which raises one of the challenges to study their function (1,7,8). Many parasitic subviral RNAs, such as defective-interfering RNAs (DI-RNAs) and satellite RNAs (satRNAs), are non-coding and accumulate to high levels in the presence of cognate helper virus (HV) in infected cells (9–12), which is a unique feature of subviral RNAs differing from cell endogenous lncRNAs. Thus, subviral RNAs are an ideal model for studying their structure and function, which can broaden our understanding of lncRNA biology.

Since satRNAs encode neither RNA replicase nor coat protein (CP), they necessarily depend on their HV for replication, encapsidation and movement. The presence of satRNAs can cause profound alterations to development of HV-induced disease symptoms. In a few cases, the HV-induced symptoms in certain host plants are intensified by a specific satRNA isolate (13–19). Turnip crinkle virus (TCV)-associated satRNA C (satC) is a well-known virulent enhancer that is a chimeric RNA composed of another smaller TCV satRNA (sat-D) at its 5′ end and the 3′ terminal portion of the TCV genomic RNA (gRNA) at its 3′ end (20). The addition of sat-C reduces TCV virion formation, leading to the presence of more free CP proteins in the infected cells (21). Since TCV CP is an RNA silencing suppressor, the increased amount of free CP thus enhances the inhibition to host RNA silencing pathways, subsequently showing the exacerbation of TCV-induced symptoms (11,21). A few cucumber mosaic virus (CMV)-associated satRNA isolates, such as sat-D4 or sat-Y, induce systemic necrosis in tomato plants (16,18). Besides the induction of necrosis in tomato, sat-Y also causes yellowing symptom in some *Nicotiana* sp., including tobacco plants (22). The underlying mechanism of yellowing was uncovered by two independent groups (23,24). They identified a sat-Y-derived small-interfering RNA (siRNA) that guides RNA silencing machinery to degrade a tobacco mRNA encoding the magnesium protoporphyrin chelatase subunit 1 involved in chlorophyll biosynthesis.

In most cases, the presence of satRNAs attenuates HV-induced disease symptoms. A typical example is CMV satRNAs that have been used as a model for investigation of the regulatory mechanism (9–12,25). CMV satRNAs are linear, single stranded lncRNA molecules with a size ranging from 332 to 405 nt (26). CMV satRNAs fully depend on CMV for replication, encapsidation, movement and transmission. CMV is an economically important plant pathogen, which is distributed worldwide. Very recently, a survey of plant viruses in over 41 000 vegetables crops samples, in the families *Solanaceae, Cucurbitaceae, Leguminosae* and *Cruciferae*, collected from 31 provinces in mainland China during 2013–2017, demonstrated that CMV is distributed in all these provinces and is the most dominant virus among 63 virus species detected in the vegetables of these four families (27). CMV has a positive-sense, single-stranded tripartite genome, designated RNAs 1–3 (26). RNAs 1 (ca. 3360 nt) and 2 (ca. 3050 nt) encode subunits 1a (methyl transferase and helicase) and 2a (RNA dependent RNA polymerase, RdRp) of the CMV replicase, respectively, but also are required for replication of satRNA, if present. RNA2 encodes an additional protein, 2b, which is translated from RNA4A (ca. 630–700 nt), the subgenomic RNA of RNA2 (28). CMV 2b has been well-characterized as an RNA silencing suppressor that inhibits siRNA-mediated antiviral defense by sequestering siRNAs, preventing siRNA entering into the RNA silencing machinery (29–32). In addition, CMV 2b has an off-target interference with host microRNA functions, which at least partially contributes to the development of CMV-induced disease symptoms (33–35). RNA3 (ca. 2220 nt) also contains two open reading frames (ORFs) encoding the 3a protein (movement protein, MP) and CP. CP is translated from the subgenomic RNA4 (ca. 1000–1250 nt) of RNA3, and it is mainly involved in viral movement and encapsidation. Recently, Zhang *et al.* (36) found that CP has the ability to negatively regulate the suppressor activity of the CMV 2b protein.

That CMV satRNAs attenuate the HV-induced symptoms has been investigated extensively. In a few specific cases, CMV satRNAs have little or no effect on virus accumulation (14,37), but they indeed attenuate the HV-induced viral symptoms, which might be due to the reduced expression level or the interference with the suppressor activity of CMV 2b protein, as proposed previously (37,38). In many cases, the attenuation of CMV-induced symptoms coincides with the reduction in the accumulation of viral RNAs (38–44). Based on the *in vitro* competition assay, Wu and Kaper (45) proposed that the reduction was due to the competition of satRNA for limited amount of viral replicase with CMV gRNAs. The competition model also fits well with other plant virus-associated satRNAs and DI-RNAs (46–49), demonstrating the generalization of this model. With the exception of several chimeric satRNA species (20,49), all satRNAs have little or no sequence similarity to their HV genome, implying that they may adopt functionally equivalent RNA structures to compete for viral and host resources with their HV. Such an RNA structure has been illustrated only in the satRNAs of bamboo mosaic virus (BaMV) (50,51). BaMV satRNA BSL6 harbors an apical hairpin stem-loop structure in its 5′ untranslated region (UTR) (50), which is responsible for the competition for replication complexes with a similar stem-loop structure in the 5′ UTR of the HV gRNA (50,51). However, the structural basis for CMV satRNA competing for replication with CMV gRNAs is totally unknown. Moreover, none of substructures of CMV satRNA has been experimentally characterized so far, although the secondary structures of some CMV satRNA isolates have been predicted by logistical programs based on the data from enzymatic or chemical probing method in several studies (52–56).

Here we examined the replication of an isolate of CMV satRNA T1 (sat-T1) that attenuated CMV-induced symptoms, accompanied with a significant reduction in the accumulation of CMV gRNAs 1 and 2, but not RNA3. In*trans* replication assays suggested that the reduced accumulation was due to the replication competition. The genetic requirement for the competition was delineated to the 3′-side hairpin in a three-way branched secondary structure that is located downstream of the center of sat-T1. The three-way branched secondary structure is designated γ-shaped structure (γSS), in which we determined the presence of a tertiary pseudoknot structure, formed by the base paring between the loop sequence and the 5′ flanking sequence of the 5′-side hairpin. This pseudoknot is essential for the viability of sat-T1 in plants, which is consistent with its evolutionary sequence conservation. The γSS including the pseudoknot is the first substructure with an important biological relevance identified in CMV satRNA so far. Taken together, a model by which satRNA downregulates CMV accumulation and symptom expression is proposed in the Discussion.

## MATERIALS AND METHODS

### Infectious clones of CMV, satRNAs and their mutants

The T-DNA-based infectious clones (pCB301-C1, C2 and C3) of CMV RNAs 1–3 (Fny strain) were generated previously (57). To construct pCB301-RNA1Δ1a lacking expression of the 1a protein, the second and third codons of the 1a ORF were mutated to stop codons in the plasmid pCB301-C1 using one-step site-directed mutagenesis (58). To construct pCB301-RNA2Δ2a2b that expresses neither 2a nor 2b protein, we first mutated the second codon to a stop codon and the second in-frame AUG to ACG in the 2a ORF using the plasmid pF209Δ2bpro as the PCR template, via one-step site-directed mutagenesis (58), generating pF209Δ2a2b. The plasmid pF209Δ2bpro is the *in vitro* transcription version of the infectious clone of CMV RNA2 with mutations to prevent 2b expression, which was described previously (59). Then the full-length cDNA of RNA2 was amplified from pF209Δ2a2b, and inserted into the binary vector pCB301 as described previously (60). To substitute the 5′ UTR in RNA3 with the 5′ UTR of RNA2, the 5′ UTR of RNA2 was amplified, and cyclized with the linearized pCB301-C3 lacking its 5′ UTR through the ligation-independent cloning (LIC) method (61), generating pCB301-RNA3u. In the same way, the 5′ UTR in RNA2 was replaced with the 5′ UTR, or the 5′ UTR with an extension of 57 nt or 120 nt of the 3a ORF of RNA3 that follows the 5′ UTR, to generate pCB301-RNA2u, pCB301-RNA2u1 or pCB301-RNA2u2, respectively. In both constructs pCB301-RNA2u1 and pCB301-RNA2u2, the start codon ATG of the 3a ORF was mutated to ACG to prevent expression of the 5′ sequence of the 3a ORF.

Sat-T1 was originally isolated from a diseased tomato plant grown in the field in Hangzhou, China. The primary sequence of sat-T1 was determined and deposited in the GenBank (GenBank No.: DQ785472). The T-DNA-based infectious clone of sat-T1 was generated through the LIC method (61). Briefly, the full-length cDNA of sat-T1 was amplified and inserted into pCB301 pre-digested by *Stu*I and *Sma*I restriction enzymes, using the LIC method (61), to generate pCB301-satT1. In the same way, we constructed the infectious clones pCB301-satD4 and pCB301-satSD for sat-D4 and sat-SD, respectively. The sat-D4 cDNA was synthesized commercially (Sangon, Shanghai), and the sat-SD cDNA was amplified from the plasmid 35S-satR (37). The plasmids pCB301-satT1, pCB301-satD4 and pCB301-satSD were used as PCR templates to generate their mutants through one-step site-directed mutagenesis (58). All mutants were verified by sequencing to ensure that only the desired mutations were introduced.

### Plant inoculation by agroinfiltration

All T-DNA infectious clones were separately introduced into *Agrobacterium tumefaciens* GV3101 using the CaCl_2_-mediated, freeze-thaw method (62). Agrobacterium-mediated plant inoculation was performed according to the protocol described previously (60). Agrobacterium cells carrying the infectious clone of RNA1, RNA2, RNA3 or satRNA (the vector pCB301 as a control) were adjusted to 0.5 OD, then mixed in an equal amounts. The mixed cells were incubated at room temperature for 3 h and infiltrated into the fourth and fifth true leaves of *N. benthamiana* plants or RDR6i transgenic plants kindly provided by Prof. David Baulcombe. Mock-inoculated plants were infiltrated with infiltration solution (10 mM MgCl_2_, 10 mM 2-(*N*-morpholino) ethanesulphonic acid and 100 μM acetosyringone). Plants were photographed at 6 days post-infiltration (dpi). Viral infection was examined through detection of progeny viral RNAs in the inoculated and/or upper leaves by northern blot hybridization analysis, as described below. All the mutants of CMV and satRNAs were sequenced to confirm the genetic stability of the introduced mutations 6 days after inoculation.

### Northern blot hybridization analysis

Total RNAs were extracted from leaf tissues with RNA extraction buffer (0.05 M NaOAc pH 5.2, 0.01 M EDTA pH 8.0 and 1% SDS), and separated by electrophoresis through 1.5% agarose gels containing 2.2 M formaldehyde. Northern blot hybridization analysis of CMV or satRNAs was performed according to the procedure described previously (32). The digoxin (DIG)-labeled DNA oligonucleotide probes for detection of either all CMV RNAs, or only RNA2 and its subgenomic RNA4A, have been described in our previous work (57). CMV RNA1-specific oligonucleotide probe is complementary to the sequence at positions 3011–3049 of RNA1. The DIG-labeled DNA oligonucleotide probe used for detection of CMV satRNAs is complementary to the sequence at nucleotide positions 35–69 of sat-T1, sat-SD or sat-D4. For detection of satRNA-derived siRNAs, 15 μg of total RNA was used for northern blot hybridization analysis according to the protocol described previously (32). Three DNA oligonucleotides corresponding to the sequences at the nucleotide positions 41–80, 141–179, 271–310 of sat-T1 were labeled with DIG at their 3′ end using the DIG oligonucleotide tailing kit generation II (Roche), and used as the probe to detect satRNA-derived siRNAs (sat-siRNAs). It is worth noting that these three oligonucleotides perfectly match to both sat-D4 and sat-SD. DIG-labeled DNA probes were detected using a chemiluminescence-based DIG detection kit (Roche) according to the manufacturer's instructions.

### Western blot analysis

Total proteins were extracted from upper systemic leaves of the infected plants using phosphate-buffered saline (0.14 M sodium chloride, 0.01 M potassium phosphate, pH 7.4) supplemented with 2% (v/v) 2-mercaptoethanol according to the procedure described previously (32). Extracted proteins were denatured in 1× Laemmli denaturation buffer (63) for 10 min, then separated by electrophoresis on SDS-containing 15% polyacrylamide gels, followed by electrophoretic transfer to nitrocellulose membranes. The membranes were incubated with polyclonal anti-CP or anti-2b serum, obtained from Prof. Xueping Zhou and Prof. Xianbing Wang, respectively, followed by incubation with a secondary antibody conjugated with horseradish peroxidase (Santa Cruz). The secondary antibody was detected with a chemiluminescence reagent kit (Thermo-Fisher) according to the manufacturer's instruction.

### RNA structure analysis

The mFold web server (64) was used to predict secondary structures for CMV satRNAs with the default parameters. To test the γSS predicted to be present in sat-T1 and sat-SD, the flexibility of these residues in this structure of sat-T1 was determined using selective 2′-hydroxyl acylation analyzed by primer extension (SHAPE) assays as previously described (65). RNA transcripts of sat-T1 were synthesized *in vitro* using a T7 High Yield RNA Synthesis Kit (NEB) according to the manufacturer's instruction. The synthesized transcripts were added to folding buffer (80 mM Tris–HCl pH 8.0, 160 mM NH_4_Cl and 11 mM Mg acetate), followed by denaturation at 75°C for 5 min, and folding at 37°C for 20 min. The folded RNAs were then chemically modified by incubation with either 17.5 mM *N*-methylisatoic anhydride (NMIA) or DMSO as a control at 37°C for 40 min. Subsequently, the modified RNAs were used as templates for reverse transcription using the primer complementary to the sequence at nucleotide positions 277–292 of sat-T1. The cDNAs synthesized were separated by electrophoresis through an 8% polyacrylamide gel containing 8 M urea. The gel was dried and exposed to a phosphorimaging screen. The intensity of each band was quantified using the semi-automated footprinting analysis software (66,67) according to the detailed protocol (67). After quantification, the effect of NMIA modification on the flexibility of each nucleotide was examined by subtracting the intensity of each band in the NMIA lane by that in DMSO, creating a net value for each nucleotide. The top 10% of the net values were selected to generate a mean value, which corresponded to the relative flexibility value of 1. Subsequently, relative flexibility values of each nucleotide were calculated by dividing their corresponding net values by the mean value. The nucleotide with a relative flexibility value of higher than 0.6 or ranging from 0.3 to 0.6 was adjudged to have a medium to high or low to medium flexibility, respectively.

## RESULTS

### Sat-T1 attenuated CMV-induced symptoms, correlated with a reduction in the accumulation of viral RNAs 1, 2 and 4A

**Figure 1.**
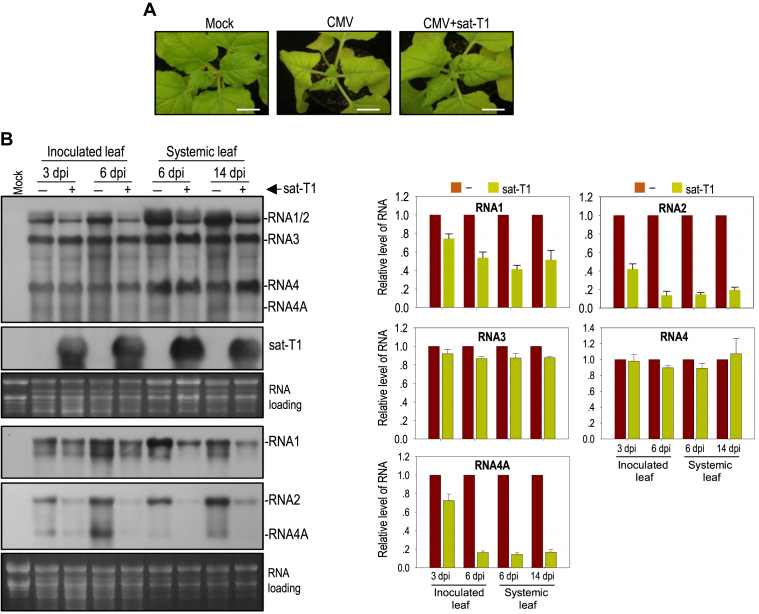
Sat-T1 attenuated viral symptoms, accompanied with a decreased accumulation of viral RNAs in plants. *Nicotiana benthamiana* plants were inoculated with CMV alone or plus sat-T1 via agroinfiltration. Mock plants were treated with infiltration solution. (**A**) Viral symptoms in *N. benthamiana* plants. The plants were photographed at 6 days post-infiltration. Bar scale, 1 cm. (**B**) Northern blot hybridization analysis of viral RNAs and sat-T1 in the inoculated or upper systemic leaves at different time points as indicated. The DNA oligonucleotide probes targeting CMV 3′ UTR, 1a and 2b were used to detect all viral RNAs, RNA1, and RNA2 and its subgenomic RNA4A, respectively. The ethidium bromide-stained rRNAs were used as a loading control. The signal intensities of viral RNAs were arbitrarily quantified using the program Gel Pro Analyzer 4.0. The relative level of each viral RNA is shown in the chart below. All of viral RNAs in the CMV-infected plants at each time point were assigned a value of 1. The columns represent the mean value and standard error from three independent biological experiments.

It has been reported previously that sat-T1 intensifies CMV-induced disease symptoms in tomato plants, including the occurrence of necrosis (43), which is akin to that caused by sat-D4 (16,18). Here, we tested the effect of sat-T1 on the expression of disease symptoms caused by a severe strain (Fny) of CMV in *N. benthamiana* plants via agroinfiltration. Viral symptoms, typically distortion of top leaves appeared at 4 dpi in the plants infected with either CMV alone or CMV plus sat-T1 (data not shown), indicating that sat-T1 did not delay the occurrence of CMV-induced symptoms. However, CMV-induced symptoms were moderately attenuated in the presence of sat-T1 early by 6 dpi (Figure 1A), demonstrating that sat-T1 is a benign satRNA in *N. benthamiana* plants.

To determine whether the attenuation of symptom expression is associated with the CMV accumulation level, we analyzed the accumulation of CMV RNAs in both the inoculated leaves at 3 dpi and 6 dpi and the upper systemic leaves at 6 dpi and 14 dpi by northern blot hybridization (Figure 1B). To accurately quantify all viral RNA segments, here, we separately used three oligonucleotide probes, which were 1a probe specific for RNA1, 2b probe specific for RNA2 and its subgenomic RNA4A, and CMV probe targeting all viral RNAs (Figure 1B). Quantification results showed that the presence of sat-T1 had a moderate effect on the accumulation of RNA1 with a reduction by 25%-60%, and dramatically reduced the accumulation of RNA2 and RNA4A by about 85% in all leaf samples, with the exception of both RNAs showing a moderate reduction in the inoculated leaves at 3 dpi (Figure 1B). However, sat-T1 had limited or no effect on the accumulation levels of RNA3 and its subgenomic RNA4 (Figure 1B). These results suggest that sat-T1 has the capability of selectively inhibiting the accumulation of viral RNAs, mainly targeting viral replicase-encoding gRNAs and the suppressor 2b-encoding RNA4A, which correlates with the attenuation of CMV-induced disease symptoms.

### Inhibition of CMV accumulation by sat-T1 was viral RNA-specific, independent of RDR6-mediated RNA silencing

Explanations for the inhibition of the accumulation of CMV RNAs by sat-T1 might be due to either increased instability or impaired replication efficiency of the viral RNAs. Zhu and colleagues (68) reported that a siRNA molecule (satsiR12) derived from sat-SD targeted RNA1 of SD-CMV for degradation, which required RDR6, but was suppressed in the presence of the CMV 2b protein. Sequence analyses show that sat-T1 also contains the same sequence corresponding to satsiR12, which suggested that sat-T1 produced the same siRNA molecule for specific degradation of CMV RNAs 1 and 2 via the siRNA-mediated silencing pathway. However, the later seemed unlikely, since the presence of the CMV 2b protein in our experiment should inhibit the satsiR12-mediated silencing of CMV RNAs (Figure 1). To determine whether the sat-T1-mediated reduction in level of CMV RNAs was RDR6-dependent, we tested the accumulation of CMV in *N. benthamiana* RDR6i plants (downregulated for RDR6 expression) in the presence or absence of sat-T1. As expected, sat-T1 had an equivalent efficiency in reducing the accumulation of CMV RNAs 1, 2 and 4A in the RDR6i plants as did sat-T1 in wild-type plants (Supplementary Figure S1), demonstrating that the inhibition of the accumulation of viral RNAs by sat-T1 was not due to satsiR12-mediated, RDR6-dependent antiviral silencing.

**Figure 2.**
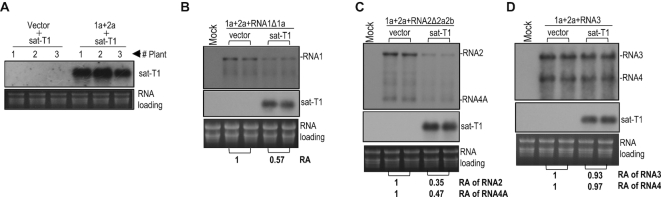
Sat-T1 inhibited the replication of a modified RNA1 (RNA1Δ1a) and RNA2 (RNA2Δ2a2b), but not RNA3. (**A**) Replication of sat-T1 promoted by the transiently expressed CMV replicase. Sat-T1 was transiently co-expressed with the CMV 1a and 2a proteins or a vector (pCB301) by agroinfiltration into the 6^th^ true leaves of *Nicotiana benthamiana* plants. RNA silencing suppressor, tomato bushy stunt virus-encoded p19 was co-expressed in the experiment. The accumulation of sat-T1 in the infiltrated leaves was analyzed by northern blot hybridization at 5 days post-infiltration (dpi). (B-D) Replication of RNA1Δ1a (lacking the 1a protein), RNA2Δ2a2b (lacking the 2a and 2b proteins) or RNA3 in the presence or absence of sat-T1 promoted by the transiently expressed CMV replicase. The CMV 1a and 2a proteins were co-expressed with RNA1Δ1 (**B**), RNA2Δ2a2b (**C**) or RNA3 (**D**), combined with or without the expression of sat-T1 via agroinfiltration as described above. The p19 silencing suppressor was co-expressed in this experiment. Viral and satellite RNAs in the infiltrated leaves were examined by northern blot hybridization at 5 dpi. Their relative levels were arbitrarily quantified and shown below. Equal loading was confirmed by staining of rRNAs with ethidium bromide.

Then, we tested the possibility that sat-T1 impaired the replication of CMV RNAs 1 and 2. Previously, we reported that the in trans CMV replication system, where CMV replicase components 1a and 2a were transiently co-expressed plus RNA silencing suppressor p19 in *trans*, supported the replication of RNA2 or RNA3 efficiently, in *N. benthamiana* plants via agroinfiltration (57). Here we first tested the replication of sat-T1 using the in*trans* replication system. Northern blot hybridization analysis of sat-T1 accumulation showed that sat-T1 was undetectable when co-expressed with a vector control, but it accumulated substantially when co-expressed with the CMV 1a and 2a proteins (Figure 2A), demonstrating that CMV replicase provided in*trans* is sufficient to replicate sat-T1. Then, we tested the effect of sat-T1 on the replication of a modified RNA1 (RNA1Δ1a) lacking expression of 1a, a modified RNA2 (RNA2Δ2a2b) expressing neither 2a nor 2b, and wild-type RNA3 using the in*trans* replication system. Northern blot hybridization analyses showed that the presence of sat-T1 reduced the accumulation level of RNA1Δ1a by 43% (Figure 2B), and it had a similar effect on the accumulation of RNA2Δ2a2b and its subgenomic RNA4A with a reduction of 65% and 53%, respectively (Figure 2C). However, sat-T1 had limited effect on the accumulation of RNA3 and RNA4 (Figure 2D). Taken together, our data suggest that the inhibition of the accumulation of CMV RNAs by sat-T1 was due to the competition of sat-T1 for replication with CMV RNAs 1 and 2, but not RNA3.

### The potential interaction of the 5′ UTR of RNA2 with sat-T1 was not the cause of the inhibition of accumulation of CMV RNA2 by sat-T1

**Figure 3.**
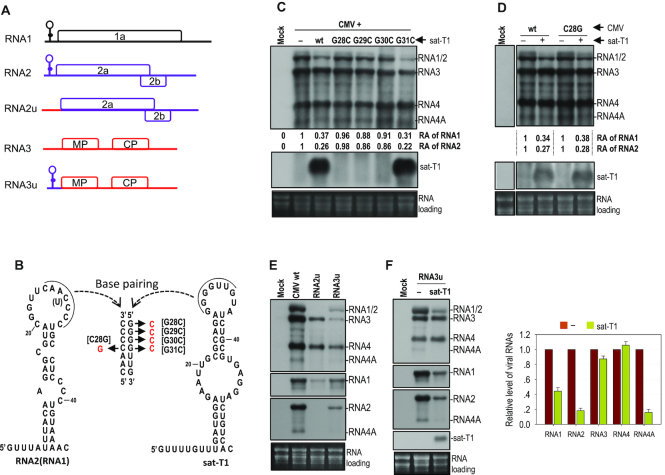
The 5′ UTR played no role in the reduced accumulation of CMV RNAs 1 and 2 caused by sat-T1. (**A**) Schematic diagrams of CMV genomic RNAs and their derivatives. An identical secondary structure shown at the 5′ end of RNAs 1 and 2 was proposed previously (69). RNA2u and RNA3u are chimeric RNA2 and RNA3, respectively, generated by exchanging their 5′ UTR between RNA2 and RNA3. (**B**) Base paring between the proposed structure in the 5′ UTR of CMV RNA2 (RNA1) and the predicted structure at the 5′ end of sat-T1. The complementary sequences in both structures are underlined. The residue in parentheses is present in RNA1. The names of the mutations are bracketed. (**C–F**) Northern blot hybridization analysis of the accumulation of CMV, sat-T1 and their mutants in the infiltrated leaves. All the mutants are shown in the panel (B). *Nicotiana benthamiana* plants were inoculated via agroinfiltration with CMV wild-type (wt) or mutant in the presence or absence of sat-T1 or its mutants. Inoculation of CMV alone is indicated as ‘-’. Total RNAs were extracted from the infiltrated leaves at 6 days post-infiltration. In panels E and F, the DNA oligonucleotide probes targeting CMV 3′ UTR, 1a and 2b were used to detect all viral RNAs, RNA1, and RNA2 and its subgenomic RNA4A, respectively. The relative accumulation levels of CMV RNAs 1 and 2 are shown below (C, D) or in the chart (F). The relative levels of RNAs 1 and 2 in the CMV-inoculated plants are assigned a value of 1. The columns shown in the chart represent the mean value and standard error from three independent biological experiments. The ethidium bromide-stained rRNAs were used as a loading control.

The 5′ leader sequence of RNAs 1 and 2 from all CMV strains are highly homologous, and can be folded into a stable hairpin structure as proposed previously (Figure 3A) (69). Such a secondary structure has been predicted at the 5′ end of CMV satRNAs (53,55,56,69), but is absent in the 5′ UTR of CMV RNA3 (Figure 3B) (69,70). These hairpin structures possessed by RNAs 1 and 2 of CMV (Fny strain) and sat-T1 are shown in Figure 3A. Interestingly, seven consecutive bases are complementary between the hairpin loop of sat-T1 and that of CMV RNAs 1 and 2, as reported previously for other CMV and satRNA isolates (10,69). It has been suggested previously that CMV satRNAs might interact directly with RNAs 1 and 2 by base paring *in vivo* via the complementary sequences to modulate the replication of CMV RNAs 1 and 2 (69). Thus, we wondered whether the inhibition of the accumulation of CMV RNAs 1 and 2 by sat-T1 is due to such base paring. Hence, single base mutations were introduced into the complementary sequence in CMV RNA2 or sat-T1 for disruption of the base paring as shown in Figure 3B. The guanine bases at nucleotide positions 28–31 in the pairing sequence _28_GGGGUUG_34_ of sat-T1 were individually mutated into a cytosine, generating four sat-T1 mutants G28C, G29C, G30C and G31C. Northern blot hybridization analyses showed that among the four mutants, only G31C survived with a similar accumulation level to that of wild-type sat-T1 in the infiltrated leaves at 3 dpi, and it markedly reduced the accumulation of CMV RNAs 1 and 2, as did wild-type sat-T1 (Figure 3C). Guanine at nucleotide position 31 in sat-T1 can potentially pair with cytosine at nucleotide position 28 in RNA2. Thus, we mutated the cytosine base at nucleotide position 28 to a guanine in the pairing sequence _25_CAACCCC_31_ of RNA2, and tested the mutant C28G in *N. benthamiana* plants at 3 dpi. We found that C28G mutant accumulated at a high level as did wild-type CMV when they were free of sat-T1 (Figure 3D). Addition of sat-T1 had an equivalent inhibition of the accumulation of RNAs 1 and 2 of this mutant and wild-type CMV (Figure 3D). All the results obtained from the mutants G31C and C28G suggested that the sequence complementarity may not mediate the intermolecular interaction between sat-T1 and RNAs 1 and 2, which is consistent with a previous report using a different approach (71).

Besides the similar hairpin structures containing the complementary sequences at their loops, sat-T1 and RNAs 1 and 2 of CMV have homologous primary sequences at their 5′ ends (5′GUUUAUUU for sat-T1, 5′GUUUUGUUUU for RNAs 1 and 2), which is absent at the 5′ end of RNA3. These common features between sat-T1 and RNAs 1 and 2 of CMV prompted us to speculate that the 5′ UTRs might be responsible for the reduced accumulation of CMV RNAs 1 and 2 caused by sat-T1. To test this, we replaced the 5′ UTR in RNA2 with the 5′ UTR of RNA3, generating a chimeric RNA2, termed RNA2u (Figure 3A), and examined the accumulation of the recombinant virus composed of RNA1, RNA2u and RNA3 in *N. benthamiana* plants. Unfortunately, the substitution of 5′ UTR was detrimental to the accumulation of RNA2u in the virus-infected local leaves at 3 dpi, but RNAs 1 and 3 both replicated, although they accumulated to a lesser extent (Figure 3E). Even when the 5′ UTR was extended beyond the 5′ terminal 120 nt of the 3a ORF, the accumulation of modified RNA2 (RNA2u1 and RNA2u2) was not improved (Supplementary Figure S2). Then, we examined another recombinant virus, in which the 5′ UTR in RNA3 was substituted with the 5′ UTR of RNA2, termed RNA3u (Figure 3A). Northern blot hybridization analysis showed that all viral RNAs of this mutant, including the modified RNA3 were detected, although their accumulation levels were much lower than those of wild-type CMV (Figure 3E). Subsequently, we examined the effect of sat-T1 on the accumulation of this mutant in the inoculated leaves of *N. benthamiana* plants at 6 dpi. Sat-T1 significantly reduced the accumulation levels of RNAs 1, 2 and 4A, but had no effect on the accumulation levels of RNA3u and its subgenomic RNA4 (Figure 3F), just as did sat-T1 to wild-type CMV (Figure 1B). These results suggest that the 5′ UTR plays no role in the reduced accumulation of CMV RNAs 1 and 2 caused by sat-T1.

### Different satRNAs had differential ability to attenuate viral symptoms and inhibit viral accumulation

**Figure 4.**
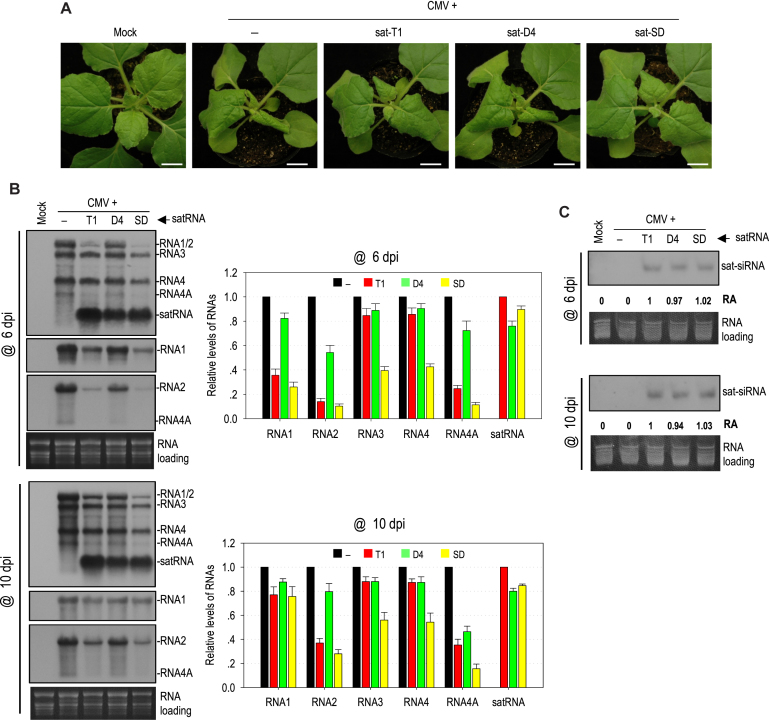
Different satellite RNAs had differential ability to attenuate symptom expression and inhibit viral accumulation. (**A**) Viral symptoms in *Nicotiana benthamiana* plants infected with CMV alone or plus satRNA T1, D4 or SD. Plant inoculation was performed via agroinfiltration, and the plants were photographed at 6 days post-inoculation (dpi). Mock plants were treated with infiltration solution. Bar: 1cm. (**B**) Northern blot hybridization analysis of the accumulation of viral RNAs and satRNAs in the upper systemic leaves at 6 and 10 dpi. The DNA oligonucleotide probes targeting CMV 3′ UTR, 1a and 2b were used to detect all viral RNAs, RNA1, and RNA2 and its subgenomic RNA4A, respectively. The signal intensities of viral RNAs were arbitrarily quantified using the program Gel Pro Analyzer 4.0. The relative level of each viral RNA was shown in the chart on the right. All of viral RNAs in the CMV-infected plants at each time point were assigned a value of 1. The columns represent the averaged value and standard error from three independent biological experiments. (**C**) Northern blot hybridization analysis of the accumulation of satRNA-derived siRNAs (sat-siRNAs) in the upper systemic leaves. The signal intensities of sat-siRNAs were arbitrarily quantified using the program Gel Pro Analyzer 4.0. The numbers shown below represent the relative accumulation levels of sat-siRNAs.

To investigate whether other CMV satRNAs have equivalent ability as sat-T1 to downregulate symptom expression and viral accumulation, sat-SD and sat-D4 with a similar size to sat-T1 were tested in *N. benthamiana* plants. Unexpectedly, both satRNAs displayed obviously differential capability of attenuating CMV-induced symptoms by 6 dpi (Figure 4A). Compared with sat-T1, sat-SD was even better at attenuating the CMV-induced symptoms. However, sat-D4 had little effect on the CMV-induced disease symptoms. Subsequently, we tested the accumulation of CMV and satRNAs in the upper systemic leaves of these infected plants at 6 dpi and 10 dpi (Figure 4B). Northern blot hybridization analyses showed that sat-SD and sat-D4 accumulated at a similar level slightly lower than that of sat-T1, but they had markedly distinct impact on CMV accumulation at 6 dpi. Sat-SD dramatically reduced the accumulation level of RNAs 1 by 74%, and RNA 2 and 4A by ∼90%, which was even more than that reduced by sat-T1. Moreover, sat-SD reduced the accumulation level of RNA3 and its subgenomic RNA4 by ∼60%, which was not observed in the case of sat-T1. In contrast, sat-D4 was a weak inhibitor to CMV accumulation, with a reduction of RNAs 1, 2 and 4A by <46%. A similar pattern of the reduction in CMV accumulation by these satRNAs was observed at 10 dpi. Meanwhile, we analyzed the accumulation of satRNA-derived siRNAs in the plants. Northern blot hybridization analysis showed no discernable difference in the accumulation of siRNAs among these three satRNAs at both time points (Figure 4C), demonstrating that the reduction in the accumulation of CMV RNAs by satRNAs was unrelated to the abundance of sat-siRNA. Collectively, all the results demonstrate that these satRNAs have differential ability to attenuate symptom expression, which coincided with the reduced accumulation levels of CMV RNAs 1 and 2, as well as RNA4A.

### Comparison of the primary sequences and predicted secondary structures of CMV satRNAs

**Figure 5.**
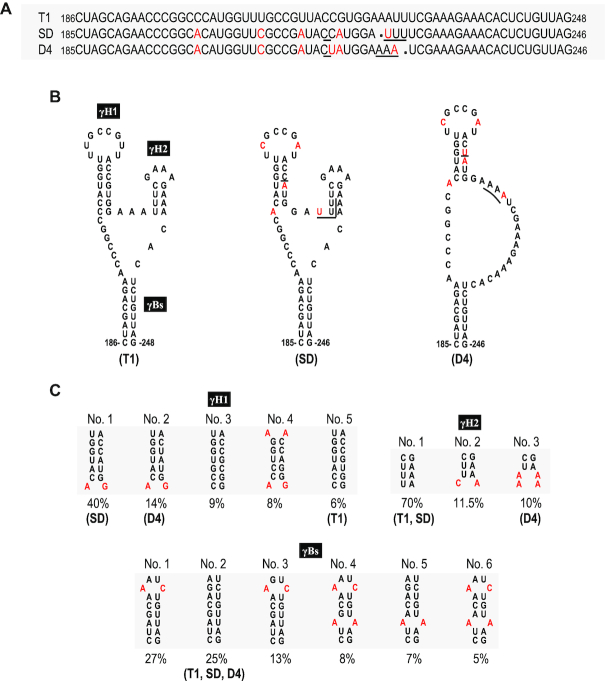
Comparison of the primary sequences and predicted secondary structures of CMV satRNAs. (**A**) Alignment of the nucleotide sequences of sat-T1, SD and D4 ranging from nucleotide positions 185–246, or 186–248. The nucleotides of sat-SD or sat-D4 that are different from sat-T1 are colored red. In total, there are four nucleotides varying between sat-SD and sat-D4 underlined. (**B**) The predicted secondary structures for these primary sequences shown in the panel (A). Sat-T1 and sat-SD can be folded into a three-way branched structure containing two hairpins (γH1 and γH2) branched from the basal stem γBs. Compared with sat-T1 or sat-SD, sat-D4 lacks the γH2 hairpin. The nucleotides colored red or underlined are the same as shown in the panel (A). (**C**) Conservation analysis of the predicted structures. All 182 sequences of CMV satRNAs deposited in GenBank were analyzed. The sequences of these three stems with a frequency of ≥5% are shown here.

To delineate the functional domain(s) of satRNAs responsible for the downregulation of CMV accumulation, we analyzed the similarity of the primary sequences among these three satRNAs tested. Sat-T1 is genetically close to sat-D4 and sat-SD with a similarity of 94.3% and 94.6%, respectively. Strikingly, sat-SD is highly homologous to sat-D4 with a 98.8% similarity, although they have considerable differences in their downregulation of viral accumulation and symptom expression. There are only four residues difference between sat-SD and sat-D4, which are located at nucleotide positions 217 and 223–225 (Figure 5A). We predicted secondary structures for these satRNAs using mFold. Interestingly, the sequence surrounding the differential residues can be folded into a three-way branched secondary structure (namely γSS) in sat-SD, as well as sat-T1 (Figure 5B). This secondary structure is composed of two hairpins (γH1, γH2) branched from a basal stem (γBs). Sat-D4 has nearly or completely identical γH1 and γBs to sat-SD, but it lacks the γH2 hairpin because the different residues at nucleotide positions 224–225 do not engage in base-paring. The residues in sat-D4 corresponding to sat-SD γH2 are located in a bulge, which is consistent with the structure predicted previously (55). The structure analysis suggested to us that γH2 might be the genetic determinant for satRNAs to downregulate CMV accumulation and symptom expression.

To analyze the conservation of the γSS, we aligned all 182 primary sequences of CMV satRNAs deposited in GenBank, and extracted all variable sequences potentially engaged in formation of these three stems in the γSS (Supplementary Table S1). With the exception of sequences on the 3′ side of γH2, all other sequences of these stems have variations. However, analyses of base paring showed that all sequence partners with a frequency of >5% pair well with a maximum of two mismatches in γH1 or γBs (Figure 5C). Moreover, the mismatch is specific in position and residue in both stems. In the case of γH2, 70% of CMV satRNAs have this structure as does sat-T1 or sat-SD, but 30% of CMV satRNAs presumably lack it, as does sat-D4 (Figure 5C). All these data indicate that the γSS is conserved in most CMV satRNA isolates.

### The predicted secondary structure was supported by the SHAPE data

**Figure 6.**
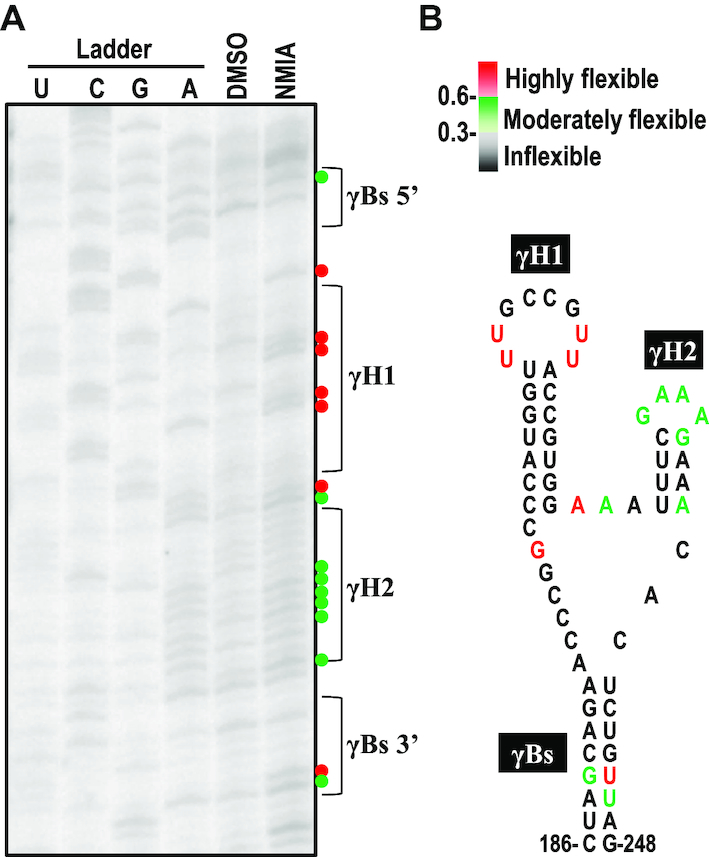
The predicted secondary structure was supported by the SHAPE data. (**A**) The SHAPE phosphorimage showing flexibility of the nucleotides in the secondary structure. G, U, C, A, nucleotide ladder lanes; D, DMSO-treated control; N, NMIA-treated. (**B**) Nucleotide flexibilities were determined using SHAPE structural probing and quantified using semiautomated footprinting analysis software. The nucleotides with high flexibility (reactivity ≥ 0.6) are denoted by a red circle or colored red, those with medium flexibility (reactivity from 0.3 to 0.6) are denoted by a green circle or colored green, and those with low to no flexibility (reactivity < 0.3) are colored black. γBs is the basal stem of the γ-shaped structure. γH1 and γH2 are the 5′ side and 3′ side hairpins, respectively.

To provide experimental evidence to support the predicted γSS, we used SHAPE structure probing methodology to investigate the flexibility of individual nucleotides in the γSS of sat-T1 *in vitro*. The phosphorimage shows the reactivity of each nucleotide to NMIA or DMSO (Control) (Figure 6A). The flexibility of each nucleotide was determined by quantifying the reactivity. The flexible nucleotides denoted by a circle in red (medium to high reactivity) or in green (low to medium reactivity), shown next to the phosphorimage, were located mainly in single-stranded regions (Figure 6B), which agrees well with the predicted structure. However, the nucleotides GCCG in the loop of γH1 and nearly all linker nucleotides between γBs and γH1 or γH2 predicted to be single-stranded were inflexible, which seems inconsistent between the predicted structure and the SHAPE data. One explanation for the inconsistencies is that these inflexible nucleotides may be involved in tertiary interactions. By comparison with the predicted secondary structures of other CMV satRNA isolates examined previously, we found that the hairpin γH1 was predicted already in several isolates, including sat-Q, -B2, -B3, -G and -WL1 (52,54), while the structures γH2 and γBs were absent in the predicted structures of all the isolates (52–56).

### A highly conserved pseudoknot was found in the γSS of sat-T1

**Figure 7.**
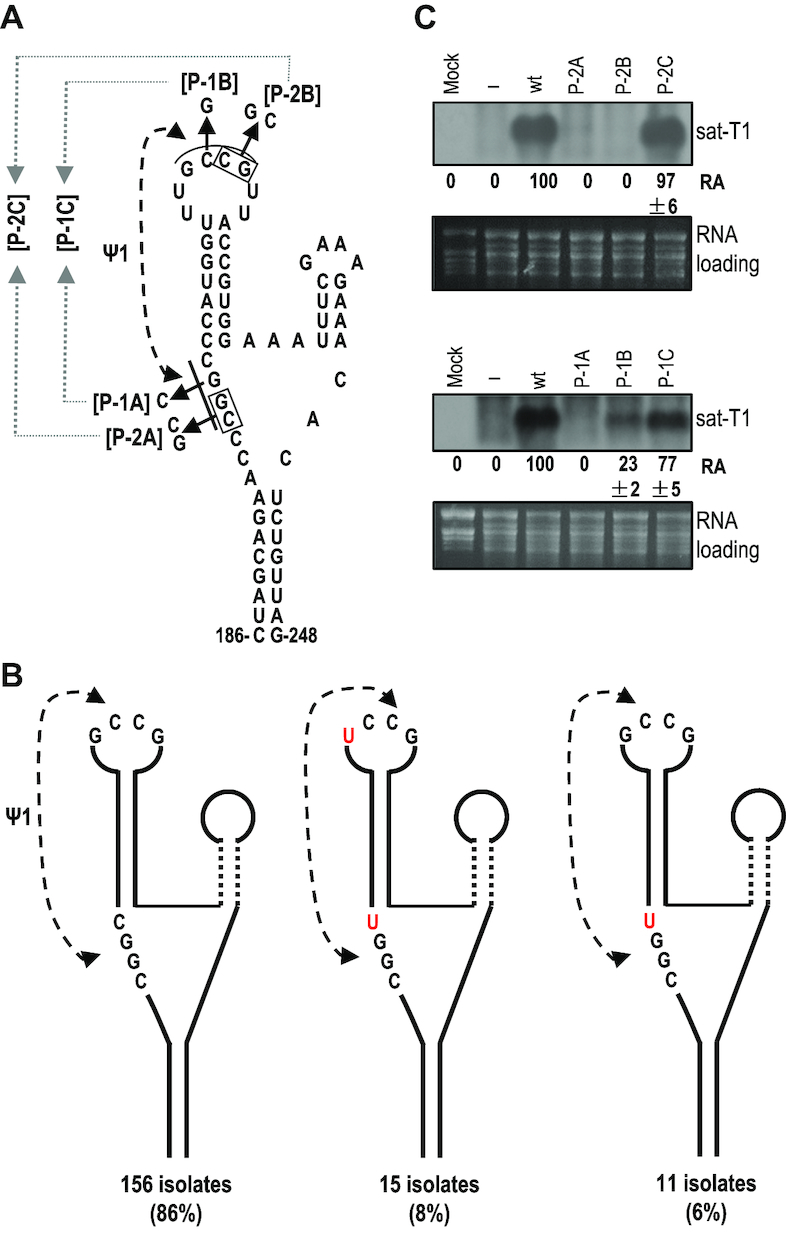
Conservation and mutational analysis of a tertiary pseudoknot in the γSS element. (**A**) A potential pseudoknot (Ψ1) interaction formed between the loop and flanking sequences of γH1. The residues assumingly engaged in the Ψ1 interaction are underlined, and their base paring is denoted by connected arrowheads. Names of the compensatory mutations generated in the interacting partners are bracketed. (**B**) Conservation of the putative Ψ1 interaction was analyzed in all 182 sequences of CMV satRNA deposited in GenBank. The residues of the pseudoknot distinct from that in sat-T1 are colored red. (**C**) Accumulation of sat-T1 with various compensatory mutations in *Nicotiana benthamiana* plants. Total RNAs were extracted from the infiltrated leaves at 3 days post-infiltration, and sat-T1 and its mutants were detected by northern blot hybridization. ‘–’ shown on the top indicates infection of CMV alone. The numbers represent the mean value and standard error from three independent experiments. The ethidium bromide-stained rRNAs were used as a loading control.

As shown above, the residues GCCG in the loop of γH1 were inflexible to NMIA, suggesting that these residues could have paring partners somewhere. Sequence analysis of sat-T1 found a potential partner CGGC located in the linker sequence between γBs and γH1 (Figure 7A), because the partner bases were single-stranded, but inflexible except for the second guanine with a high flexibility (Figure 6B). Through sequence analyses of all CMV satRNAs, we found that the potential tertiary structure termed pseudoknot1 (Ψ1) is completely conserved (Figure 7B). However, the pseudoknot was not predicted in the CMV satRNA isolates examined previously (52–56). To verify the pseudoknot and address its role, compensatory mutation analysis of the partner sequences was performed (Figure 7A). Sat-T1 with various mutations was co-inoculated with CMV into *N. benthamiana* plants via agroinfiltration, and their accumulation in the inoculate leaves was analyzed by northern blot hybridization at 3 dpi. When the mutations targeted two base pairs in the partner sequences, the disruptive mutations (P-2A, P-2B) abolished the accumulation of sat-T1, while the restoration of the base pairing (P-2C) recovered accumulation levels to that of wild-type sat-T1 (Figure 7C). The mutations targeting another base pair, substitution of the 3′ guanine residue (highly flexible) in CGGC with a cytosine (P-1A), was detrimental to the accumulation of sat-T1, while the substitution of the pairing base cytosine in the partner sequence with a guanine (P-1B) reduced accumulation levels to 23% of wild-type sat-T1. When combining both substitutions together, the mutant (P-1C) recovered accumulation levels to 77% of wild-type sat-T1 (Figure 7C). Taken together, these results provide strong evidence to verify the tertiary pseudoknot structure in the predicted γSS, and demonstrate its important role in satRNA survival.

### Functional analysis of the γSS element

**Figure 8.**
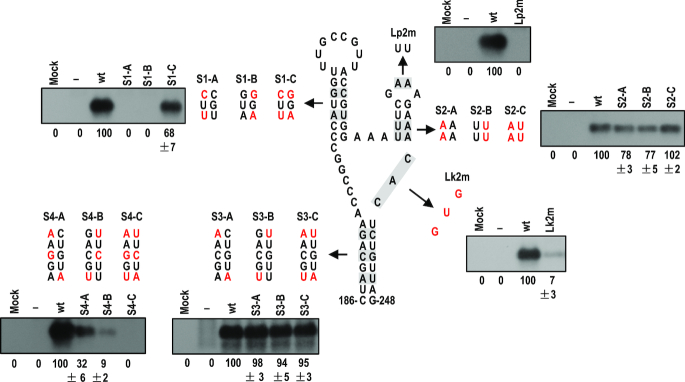
Mutational analysis of the γSS element. Residues in the structure that were targeted for mutation are shaded and the residues mutated in corresponding mutants are colored red. Northern blot hybridization analysis was used to determine the accumulation of sat-T1 and its mutants in the inoculated leaves at 3 days post-infiltration. ‘–’ shown on the top indicates infection of CMV alone. The values represent the mean percentages of relative accumulation levels from three independent experiments with standard errors. The ethidium bromide-stained rRNAs were used as a loading control.

After identification of the novel pseudoknot in the predicted γSS, we analyzed the biological function of the predicted γSS by introducing compensatory mutations into the stems or complementary nucleotides into some unpaired residues (Figure 8). All mutants were tested in the inoculated leaves of *N. benthamiana* plants at 3 dpi. Disruption of the γH1 stem by the mutation of two base pairs (S1-A, S1-B) was lethal to sat-T1, while the restoration of the base pairing (S1-C) recovered accumulation level to 68% of wild-type sat-T1 (Figure 8). This result is consistent with the involvement of its loop in the formation of Ψ1 (Figure 7). For the stem of γH2, the disruptive mutations (S2-A, S2-B) had a moderate effect on the accumulation levels of sat-T1 with a reduction by about 20%. Restoration of the base pairing by the compensatory mutation (S2-C) increased accumulation levels to that of wild-type sat-T1 (Figure 8). For the loop of γH2, substitution of the first two adenine residues with two uridines (Lp2m) abolished the accumulation of sat-T1 (Figure 8). These results obtained from the mutations to γH2 demonstrated that the γH2 structure was not essential for the accumulation of sat-T1, but the loop sequence GAAA was extremely important for sat-T1 survival. The single-stranded residues CAC between γBs and γH2, shown to be inflexible (Figure 7), were very important to the accumulation of sat-T1, since the substitution of these residues with the complementary bases GUG reduced accumulation levels by 93% (Figure 8). As shown in Figure 5C, the basal stem γBs is highly conserved, except for two pairs of residues mismatched in some satRNA isolates. Firstly, the two base pairs in sat-T1 γBs corresponding to the mismatched residues were targeted for compensatory mutation analysis (mutant series S3) (Figure 8). As expected, all the mutations were neutral to the accumulation of sat-T1. Then, an extra base pair that is completely conserved was added for further compensatory mutation analysis (mutant series S4) (Figure 8). Both disruptive mutations (S4-A and S4-B) significantly reduced the accumulation of sat-T1 by 68% and 91%, respectively. Unexpectedly, the compensatory mutation (S4-C) was lethal to sat-T1, suggesting that the nucleotide sequence but not the structure of γBs is important for sat-T1 accumulation, which is consistent with their sequence conservation. In general, the results of functional analyses are in agreement with the predicted γSS, and the γSS plays an indispensable role in sat-T1 viability in plants.

### The γH2 hairpin is the genetic determinant for sat-T1 to downregulate viral symptoms and accumulation

**Figure 9.**
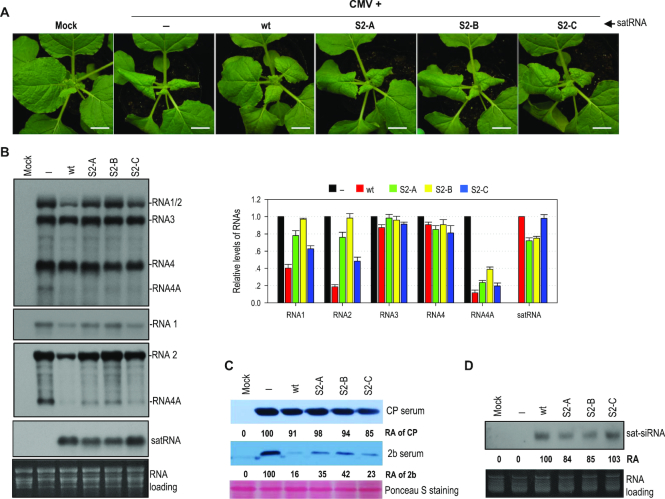
The γH2 hairpin engaged in the inhibition of the accumulation of CMV RNAs by sat-T1. (**A**) Disease symptoms in *Nicotiana benthamiana* plants infected with CMV alone (–) or plus sat-T1 wild-type (wt) or γH2 mutants as indicated on the top. These mutants have been depicted in Figure 8. The plants were photographed at 6 days post-infiltration (dpi). Mock plants were infiltrated with infiltration solution. Bar: 1 cm. (**B**) Accumulation of CMV, sat-T1 and its mutants in the infected plants analyzed by northern blot hybridization. Total RNAs were extracted from the upper systemic leaves at 6 dpi. The DNA oligonucleotide probes targeting CMV 3′ UTR, 1a and 2b were used to detect all viral RNAs, RNA1, and RNA2 and its subgenomic RNA4A, respectively. The signal intensities of CMV and satellite RNAs were arbitrarily quantified using the program Gel Pro Analyzer 4.0. The relative level of each RNA species is shown in the chart on the right. All of viral RNAs in the CMV-infected plants were assigned a value of 1, as well as wt sat-T1. The columns represent the averaged value and standard error from three independent biological experiments. The ethidium bromide-stained rRNAs were used as a loading control. (**C**) Immunoblot analysis of the accumulation of the CMV CP and 2b proteins in the infected plants. Total proteins were prepared from the upper systemic leaves at 6 dpi, and separated in a SDS-contained polyacrylamide gel for immunoblotting using antiserum against CMV CP or 2b. The values represent relative accumulation levels. (**D**) Northern blot hybridization analysis of the accumulation of satRNA-derived siRNAs (sat-siRNAs) in the upper systemic leaves at 6 dpi. The signal intensities of sat-siRNAs were arbitrarily quantified using the program Gel Pro Analyzer 4.0. The numbers shown below represent the relative accumulation level of sat-siRNAs.

As suggested above, γH2 might be the genetic determinant for downregulation of CMV accumulation and symptom expression. To address the role of γH2, we tested the γH2 mutant series (S2-A to C, shown in Figure 8) in *N. benthamiana* plants. By 6 dpi, CMV-infected plants expressed severe disease symptoms, which were reversed extensively in the presence of wild-type sat-T1 (Figure 9A). Both mutants S2-A and S2-B, with a disrupted stem, accumulated to ∼75% of wild-type sat-T1 (Figure 9B, C), but they showed much less ability to attenuate CMV-induced symptoms (Figure 9A), suggesting that the hairpin structure is required for the inhibition of viral symptoms. Interestingly, the mutant S2-C, containing the compensatory mutation, recovered accumulation levels to that of wild-type sat-T1 (Figure 9B), but it was much weaker than wild-type sat-T1 in the attenuation of viral symptoms (Figure 9A), suggesting that the attenuation of viral symptoms by sat-T1 depends not only on the hairpin structure but also the primary sequence of γH2.

Next, we analyzed the accumulation of viral RNAs in the upper systemic leaves of these plants by northern blot hybridization with the CMV-specific probe. All these mutants, akin to sat-T1, had limited or no effects on the accumulation of RNAs 3 and 4, but displayed differential inhibition to RNAs 1 and 2 (Figure 9B). The two disruptive mutants S2-A and S2-B had a nearly identical accumulation level (Figure 9B), but they exhibited differential ability to inhibit the accumulation of RNAs 1 and 2. S2-A reduced the accumulation levels of the two viral gRNAs by ∼24%, but S2-B completely lost such an inhibitory ability (Figure 9B). This demonstrates that the two residues AA themselves, at the 3′ side of the γH2 stem, are required for sat-T1 to function as an inhibitor of virus accumulation. Compared with S2-B, S2-C, containing the compensatory mutation, partially recovered the inhibition of the accumulation of both gRNAs 1 and 2, indicating that the hairpin structure also played a role in the inhibition. Moreover, the relative lower efficiency of S2-C versus wild-type sat-T1 in the inhibition further demonstrates the importance of the primary sequence of γH2. Using the 2b-specific probe, northern blot hybridization analysis clearly showed that sat-T1 and its mutants reduced the level of RNA4A to varied extents, which displayed a similar pattern to that of RNA 1 or 2, while their inhibition of RNA4A was greater than that of RNA 1 or 2 (Figure 9B). It is noteworthy that although S2-B showed no ability to inhibit the accumulation of RNAs 1 and 2, it still significantly reduced the accumulation level of RNA4A. The relative accumulation levels of RNA4A or RNA4 were consistent with the result of immunoblot analysis of CMV 2b or CP protein, respectively (Figure 9C). We also analyzed the accumulation level of sat-siRNAs in the plants (Figure 9D). Northern blot hybridization analysis showed that the accumulation of sat-siRNAs derived from sat-T1 wild-type and the mutants (S2-A, B, C) had an identical pattern as that of these satRNAs, and was not associated with the accumulation levels of CMV RNAs 1 and 2. Taken together, the γH2 hairpin, including its structure and specific residues, was the genetic determinant of sat-T1 required for downregulation of viral symptoms and accumulation.

**Figure 10.**
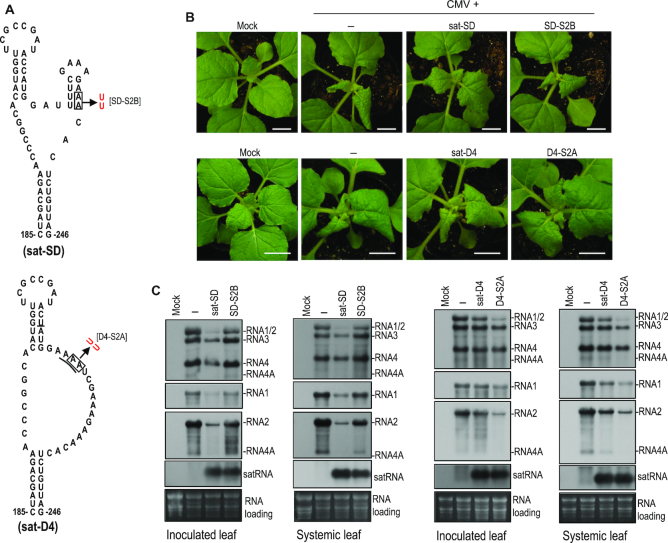
Biological relevance of the mutations that disrupted or generated the γH2 hairpin in sat-SD or sat-D4, respectively. (**A**) The γSS structure of sat-SD and its disruptive mutation to γH2 (top), and the equivalent structure of sat-D4 and its mutation (bottom). The mutation introduced to sat-D4 presumably generates a γH2 hairpin in sat-D4. (**B**) Disease symptoms in *Nicotiana benthamiana* plants infected with CMV alone (–) or plus a satRNA or its mutant as indicated on the top. The plants were photographed at 6 days post-infiltration (dpi). Mock plants were infiltrated with infiltration solution. Bar: 1 cm. (**C**) Northern blot hybridization analysis of the accumulation of CMV, satRNA or satRNA mutants in the infected plants. Total RNAs were extracted from the inoculated leaves or upper systemic leaves at 6 dpi. The DNA oligonucleotide probes targeting CMV 3′ UTR, 1a and 2b were used to detect all viral RNAs, RNA1 and RNA2 and its subgenomic RNA4A, respectively. The ethidium bromide-stained rRNAs were used as a loading control.

To further elucidate the role of γH2, we tested a sat-SD mutant, SD-S2B in which the γH2 structure is disrupted by substitution of the AA residues on the 3′ side of the stem with two uridines (Figure 10A), resembling the mutant S2-B of sat-T1 (Figures 8 and 9). Disruption of the γH2 structure had limited effects on the accumulation of sat-SD in either the inoculated or systemic leaves at 6 dpi (Figure 10C), but obviously impaired the attenuation of CMV-induced symptoms (Figure 10B), and substantially released the inhibition to the accumulation levels of RNAs 1 and 2 (Figure 10C). Moreover, in the plants infected by CMV and SD-S2B, the accumulation of RNAs 3 and 4 also recovered to the levels of those in the CMV-infected plants (Figure 10C). In the same way, we tested a sat-D4 mutant, D4-S2A, in which a γH2 structure would be formed by substitution of the adenine residues at nucleotide positions 224 and 225 with two uridines (Figure 10A). D4-S2A accumulated at a similar level to sat-D4 in the inoculated or upper systemic leaves at 6 dpi (Figure 10C), but it was remarkably better than sat-D4 in the attenuation of CMV-induced symptoms (Figure 10B), and the inhibition of the accumulation of CMV RNAs (Figure 10C). All the results obtained from both mutants further support the functional relevance of γH2.

## DISCUSSION

In this work, we found that sat-T1 specifically reduced the accumulation of CMV RNAs 1 and 2, as well as RNA4A, leading to the attenuation of viral symptoms in *N. benthamiana* plants. The structural basis for sat-T1 to reduce the accumulation of CMV RNAs was determined to be the identified γSS, which is composed of two hairpins γH1 and γH2 branched from a basal stem γBs. The γH1 hairpin engaged in formation of a pseudoknot, which is essential for sat-T1 viability. The γH2 hairpin is the genetic determinant of sat-T1 responsible for the reduction in the accumulation of CMV RNAs 1 and 2. Since the γSS element is conserved in a large portion of CMV satRNAs, it could be a generic method for CMV satRNA to inhibit the accumulation of the HV.

The γSS is the first substructure of CMV satRNA experimentally determined to be associated with an important biological phenotype. It is interesting that the γSS contains the pseudoknot Ψ1. Ψ1 was predicted to be totally conserved in all CMV satRNAs (Figure 7B), which is in agreement with its crucial role in satRNA viability (Figure 7C). To our knowledge, Ψ1 is the first tertiary structure identified in CMV satRNA so far. Pseudoknots also have been identified in a few satellite RNAs associated with other plant viruses, such as TCV and cymbidium ringspot virus (72–74). All of these pseudoknots are important for the accumulation of these satRNA species, except for Ψ3 in TCV satC (73). Ψ2 in TCV satC acts as a structural switch for activation of minus-stranded synthesis (75). The molecular basis by which other pseudoknots regulate the accumulation of plant virus satRNAs remains to be investigated.

SHAPE reagents, such as NMIA are thought to modify the 2′ hydroxyl in unpaired nucleotides. Our results demonstrate the base paring between _199_G and _211_C in Ψ1 (mutant series P-1, Figure 6), but _199_G is highly modifiable by NMIA, implying that the base paring in Ψ1 arranges the guanine residue in a way to allow its 2′ hydroxyl to be highly accessible to NMIA. Such a hypermodifiable guanine actually involved in base paring in a pseudoknot structure has been reported in the pea enation mosaic virus 2-encoded PTE structural motif, which is a translation initiation factor eIF4E-binding, 3′ cap-independent translation enhancer (76,77). Based on biochemical and bioinformatics analyses, they found that the hypermodifiable guanine protruded from the pseudoknot and docked in the cap-binding pocket of eIF4E, which is required for the binding of eIF4E to the viral RNA and subsequent enhanced translation (76). Accordingly, it is possible that Ψ1 in γSS has the ability to bind viral or host proteins, which are required for satRNA replication.

The γSS contains two hairpins γH1 and γH2, which branched from the basal stem γBs. Sequence analyses show that γBs is highly conserved, except for two base pairs (Figure 5C). Compensatory mutation analyses suggest that γBs might be non-functional in the accumulation of sat-T1 (mutant series 4, Figure 8). Such effects are also observed in other plant virus-associated satRNAs, such as sat-Cym (72). γBs might contribute to a biological process that could not be monitored in our assays. Du *et al.* (78) reported that sat-SD-derived siRNAs were generated mainly from flexibly structured single-stranded molecules. A sat-SD-derived siRNA (satsiR12) can target RNA1 of SD-CMV for degradation *in vivo* (68). Coincidently, satsiR12 has 15 nt overlapping with the 5′ terminal sequence (including the 5′ adjacent sequence of γBs) of sat-SD γSS. Combined with the fact that sat-T1 has an identical γBs to sat-SD, this allows us to propose that γBs may contribute to the biogenesis of satsiR12 *in vivo*. Thus, future comprehensive functional analyses will be required to precisely investigate the function of γBs.

Our data show that the attenuation of viral symptoms in the presence of sat-T1 is associated with the reduction in the level of RNAs 1 and 2, as well as RNA4A and its product, the 2b protein (Figures 1 and 9). Since CMV2b is a RNA silencing suppressor that mediates viral pathogenicity, the reduction in the expression of CMV 2b protein would contribute to the compromised viral symptoms, which has been demonstrated previously (37). Interestingly, the plants co-infected with CMV and the sat-T1 mutant S2-B show nearly the same severe symptoms and viral accumulation as the CMV-infected plants, except for the lower accumulation of RNA4A and the 2b protein (Figure 9), suggesting that the reduction in the level of RNAs 1 and 2 by sat-T1 was also required for the amelioration of CMV-induced symptoms. This is consistent with the previous finding that CMV lacking the functional 2b protein is pathogenic in *Arabidopsis thaliana* plants with a deficiency in antiviral silencing (29,79). To our surprise, SD-sat was a strong inhibitor of CMV accumulation and disease symptoms in our experiments (Figure 4), but it was not in a previous report (37). One explanation for the difference can be the different CMV strains used, since the effect of satRNA on CMV accumulation and symptoms depends on the triple interaction among virus strains, satRNA isolates, and host species (10,39).

Previously, Zhu *et al.* (68) reported a new mechanism for satRNAs to reduce the accumulation of CMV RNAs via targeting viral RNAs by a satRNA-derived siRNA for degradation, but which requires host RDR6 and is suppressed in the presence of CMV 2b. Our results demonstrate that the inhibition by sat-T1 of the accumulation of CMV RNAs is independent on RDR6, and cannot be inhibited in the presence of the CMV 2b protein, suggesting that this mechanism is not engaged in the inhibition of the accumulation of CMV RNAs by sat-T1. Actually, the in*trans* replication assays suggest that sat-T1 specifically impairs the replication of CMV RNAs 1 and 2, leading to reduction in their accumulation levels during virus infection (Figures 1 and 2). However, we cannot rule out the possibility that satRNA-derived siRNAs non-specifically bind to and saturate the suppressor p19 used in the replication assay, leading to enhanced RNA silencing against viral RNAs, as proposed by Shen and colleagues (38). Such a proposal was suggested from the earlier work on the inhibition of tomato bushy stunt virus accumulation by its DI-RNAs (80). However, this possibility can be argued against by the evidence that the accumulation of RNA3 is not reduced in the replication assay (Figure 2D), and no relationship exists between the abundance of sat-siRNAs and the inhibition of the accumulation of CMV RNAs 1 and 2 by satRNAs (Figures 4C and 9D).

**Figure 11.**
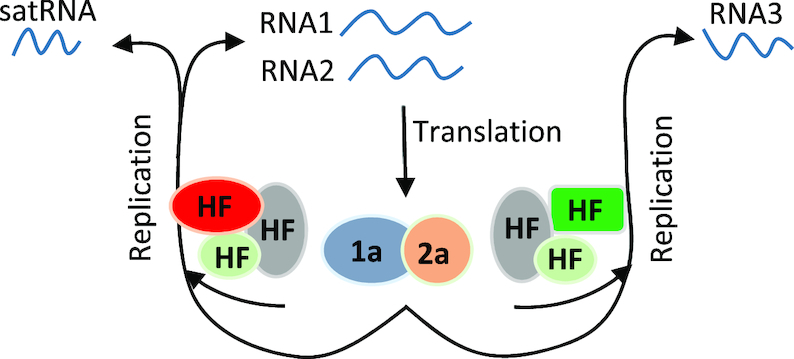
A proposed model for satRNA inhibiting the accumulation of CMV RNAs. RNAs 1 and 2 released from infected CMV are used as translation templates to produce viral replicase components 1a and 2a, respectively. Differential host factors are subsequently recruited by viral replicase and RNAs to form replication machinery for replication of RNAs 1 and 2 or RNA3. CMV satRNAs specifically compete the replication machinery for replication of RNAs 1 and 2, by which it reduces the accumulation of both viral RNAs. Once the accumulation of RNAs 1 and 2 is reduced to a level at which the 1a and 2a proteins produced by both viral RNAs become limited for RNA3 replication, the accumulation of RNA3 is reduced as observed in the case of sat-SD shown in this study.

Based on the discussion above, sat-T1 inhibits the accumulation of CMV RNAs 1 and 2 most probably by competition with CMV RNAs 1 and 2 for replication resources that can be either viral replicase, host factors or both. Competition for replicase has been proposed as a generalized model for plant subviral RNAs to reduce HV accumulation (45). *In vitro* replication assays have demonstrated the advantage of satRNA over all CMV gRNAs in competition for viral replicase (45). However, we did not observed the inhibition of the replication of RNA3 by sat-T1 (Figure 2D), suggesting that although viral replicase is required for replication of sat-T1, it might be not the limiting factor to be competed between sat-T1 and CMV RNAs. Thus, we speculate that the limiting source is the host factor(s) specifically required for replication of CMV RNAs 1 and 2, but not for RNA3 replication, as shown in the model we proposed (Figure 11). The model can be supported by the earlier work that sat-Q outcompetes CMV for host bromo-domain containing protein 1 that is involved in CMV replication (81). As the genetic determinant, γH2 would be responsible for the competition for host factor(s) that remains to be determined. The limited or lack of effect on accumulation of RNA3 and its subgenomic RNA4 by satRNAs may also be attributed to differences in requirements for replication by different CMV RNAs. Defective RNA 1 molecules cannot be replicated in *cis* (82,83). The same may be true for RNA2, but this has not been examined. Both RNAs 1 and 2 contribute replicase components. By contrast, RNA3, which does not, is replicated in *trans* (45,57,83,84). In addition, the 1a and 2a proteins are involved in the replication of different polarity RNAs and show temporal changes in replication of positive and negative strands (83). This may involve differences in host factors, the phosphorylation status of the 2a protein (83,85), or the interaction of the 2a and 3a proteins (86,87).

The γH2 hairpin is predicted to be present in 70% of CMV satRNAs (Figure 5). In the case of sat-T1, γH2 is the genetic determinant responsible for the reduction in the accumulation of CMV RNAs 1 and 2, as well as RNA4A (Figure 9). However, in some cases reported (38,39,41,43), satRNAs reduced the accumulation of not only CMV RNAs 1, 2 and 4A, but also RNAs 3 and 4, as did sat-SD in this work (Figure 4). Interestingly, disruption of γH2 in sat-SD led to partial recovery of the accumulation levels of CMV RNAs 1 and 2, but full recovery of the accumulation levels of RNAs 3 and 4 (Figure 10C). This result demonstrates that besides the γH2 hairpin, at least some satRNAs including sat-SD may have additional genetic determinant(s) or mechanism(s) involved in the inhibition of the accumulation of CMV RNAs 1 and 2 (38,68), and suggest that the reduction in the accumulation of RNA3 and RNA4 is attributed to the dramatic reduction in the levels of CMV RNAs 1 and 2, which provides insufficient replicase for RNA3 replication, accompanied with the reduced RNA4 transcription. The suggestion can be supported by the work that CMV RNA1-transgenic tobacco, which constitutively expressed limited amounts of the 1a protein and was inoculated with CMV RNA2 and RNA3, yielded much less RNAs 2–4 than wild-type tobacco infected with all three CMV RNAs (84,88).

In conclusion, we determined the presence of the biologically relevant structure γSS in CMV sat-T1. This structure contains the genetic determinant γH2 that is specifically responsible for the inhibition of the accumulation of CMV RNAs 1 and 2, presumably by competition for host factor(s) required for the replication of both viral RNAs. Since γH2 is predicted to be present in 70% of CMV satRNAs, it may be a generic method for CMV satRNAs to inhibit virus accumulation and attenuate viral symptoms. Our finding uncovers the structural basis of satRNA required for downregulating the replication of CMV gRNAs, and provides a foundation for further insights to the replication competition between satRNA and cognate HV.

## Supplementary Material

gkz564_Supplemental_File
