# Supplementary material for: A conserved RNA structure is essential for a satellite RNA-mediated inhibition of helper virus accumulation
Source: Nucleic Acids Res. 2019 Jul 3;47(15):8255–71. doi: 10.1093/nar/gkz564 (PMC6735963; doi:10.1093/nar/gkz564)
Supplement: gkz564_Supplemental_File [file gkz564_supplemental_file.pdf]

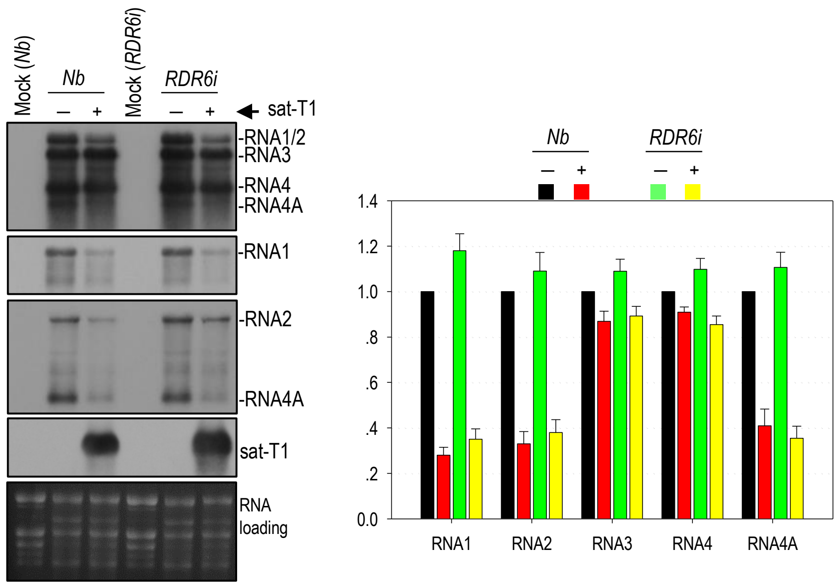

FIG S1. RDR6 was not required for sat-T1 to inhibit the accumulation of CMV RNAs 1 and 2 in *Nicotiana benthamiana* plants. CMV alone or with sat-T1 was inoculated into *N. benthamiana* (*Nb*) or RDR6i plants via agroinfiltration. Total RNAs were extracted from the inoculated leaves at 6 days post-infiltration, and the accumulation of CMV and sat-T1 was examined by northern blot hybridization. The DNA oligonucleotide probes targeting CMV 3′ UTR, 1a and 2b were used to detect all viral RNAs, RNA1, and RNA2 and its subgenomic RNA4A, respectively. The arbitrary signal intensities of the viral RNA bands were quantified using the program Gel pro 4.0, and the relative levels of the viral RNAs were calculated and shown in the chart on the right. The level of each viral RNA detected in the sat-T1-free, *Nb* plants was assigned a value of 1. Ethidium bromide-stained rRNAs were used as a loading control.

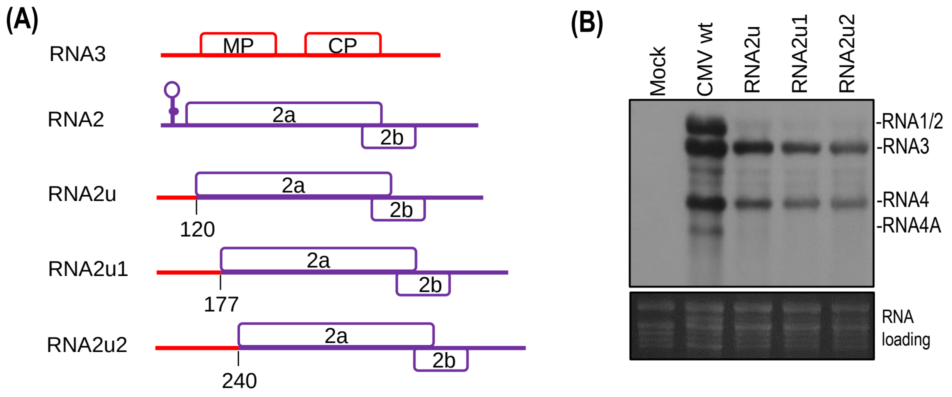

FIG S2. Replacement of the 5' UTR in RNA2 with the 5' terminal sequence of RNA3 at different length was detrimental to the replication of the modified RNA2s. (A) Schematic diagrams of CMV RNA3, RNA2 and its derivatives. RNA2u, RNA2u1 and RNA2u2 were generated by replacing the 5' UTR in CMV RNA2 with the 5' 120 nt (exactly 5' UTR), 177 nt and 240 nt of CMV RNA3, respectively. The start codon AUG of the 3a ORF was mutated to ACG in the constructs RNA2u1 and RNA2u2. (B) Northern blot hybridization analysis of the accumulation of CMV wt and its RNA2 mutants in *Nicotiana benthamiana* plants at 3 days post-infiltration. The ethidium bromide-stained rRNAs were used as a loading control.

Table S1 Sequence analysis of these three stems in the predicted γ-shaped structure from 182 CMV satRNA sequences deposited in GenBank.

	yBs ¥				yH1 ¥				yH2			
Seq No.	5' sequence	3' sequence	Upb Ω	Frequency	5' sequence	3' sequence	Ubp Ω	Frequency	ARE including yH2 5' sequence	3' sequence	Form yH2 structure?	Frequency
1	CUAGCAAA	UCUGUUAG	1	49 (27%)	ACAUGGU	ACCAUGG	1	73 (40%)	UUUC	GAAA	Y	127 (70%)
2	CUAGCAGA	UCUGUUAG	0	46 (25%)	ACAUGGU	ACUAUGG	1	25 (14%)	CUUC	GAAA	N	22(11.5%)
3	CUAGCAAG	UCUGUUAG	1	24 (13%)	CCGUGGU	ACCGCGG	0	17 (9%)	AAUC	GAAA	N	18 (10%)
4	CUAGCAAA	UCUGUAAG	2	14 (8%)	ACCUGGA	ACCAGGG	2	14 (8%)	AUUC	GAAA	N	7 (4%)
5	CUAACAGA	UCUGUAAG	1	13 (7%)	CCAUGGU	ACCGUGG	0	11 (6%)	AAUC	GAAC	N	4 (2%)
6	CUAACAAA	UCUGUAAG	2	9 (5%)	CCAUGGU	ACCAUGG	0	7 (4%)	AUUC	GAAC	N	3 (1.5%)
7	CUAGCAGA	UCUGUAAG	1	8 (4%)	UCGCGGU	ACCUCGG	1	6 (3%)	UCUC	GAAA	N	1 (0.5%)
8	CUAACAAA	UCUGUUAG	1	4 (2%)	ACAUGGU	ACCUUGG	2	5 (3%)				
9	CUAGCAAG	UCUGUAAG	2	4 (2%)	UCGCGGA	ACCUCGG	2	4 (2%)				
10	CUAGCAGG	UCUGUUAG	0	3 (1.5%)	CCAUGGU	ACCACGG	1	4 (2%)				
11	CUAACAGA	UCUGUUAG	0	2 (1%)	UCGGGGU	ACUACCG	2	3 (1.5%)				
12	CUAGCAGA	UCUGUUAU	1	1 (0.5%)	ACAUGGU	CCAUGGU	2	2 (1%)				
13	CAUGCAGA	UCUGUUAG	2	1 (0.5%)	ACGUGGU	ACCACGG	1	2 (1%)				
14	CCAGCAGA	UCUGUUAG	1	1 (0.5%)	ACAUGGU	ACCGUGG	1	1 (0.5%)				
15	CUAACAAG	UCUGUAAG	2	1 (0.5%)	CCGUGGU	UCCGCGG	1	1 (0.5%)				
16	CUAACAGA	UCUGUAUG	2	1 (0.5%)	CCAUGGU	ACCUGG	1	1 (0.5%)				
17	CUAACAGA	UCUGCAUG	3	1 (0.5%)	GCAUGGU	ACCAUGG	1	1 (0.5%)				
18					ACAUGG	ACCAUGG	2	1 (0.5%)				
19					GCGGAGU	ACUACCG	2	1 (0.5%)				
20					GCGGAGU	ACUUCCG	2	1 (0.5%)				
21					GCAGAGU	ACUUCCG	3	1 (0.5%)				
22					ACCUGGA	ACUUGGG	3	1 (0.5%)				

¥ Bases unpaired color red. Ω Ubp means the number of unpaired bases.
